# Optimal fermentation of Shuanghuanglian and its effects on production performance of laying hens

**DOI:** 10.3389/fvets.2024.1415232

**Published:** 2024-10-03

**Authors:** Yongqing Xu, Siyu Yi, Xiaojie Xu, Minghui Zhang, Yadong Cui, Wei Lan, Fenglan Li, Xiangfeng Kong

**Affiliations:** ^1^College of Life Science, Northeast Agricultural University, Harbin, China; ^2^Hunan Provincial Key Laboratory of Animal Nutritional Physiology and Metabolic Process, National Engineering Laboratory for Pollution Control and Waste Utilization in Livestock and Poultry Production, Institute of Subtropical Agriculture, Chinese Academy of Sciences, Changsha, China; ^3^School of Biology and Food Engineering, Fuyang Normal University, Fuyang, China

**Keywords:** bioactive compounds, egg quality, optimized fermentation, response surface methodology, Chinese medicine

## Abstract

**Background:**

Shuanghuanglian is a Chinese medicine composed of *Honeysuckle Flower*, *Baical Skullcap Root*, and *Fructus Forsythiae*. It has various effects, including anti-inflammatory, antibacterial, antiviral, and immunomodulatory effects. The fermented product of Shuanghuanglian can be used as an antibiotic alternative, as it has similar efficacy, which may improve the immunity, feed intake and utilization efficiency of laying hens, thus improving their production performance. The aim of this study was to optimize the fermentation conditions for Shuanghuanglian using single factor and response surface methodology, evaluate the chemical and microbial composition of the Shuanghuanglian fermentation liquor (SFL), and explore the effects of SFL on the production performance of laying hens.

**Methods:**

A total of 288 Xinyang black-feather laying hens (50 week-old) were randomly allocated to four treatments with nine replicates, each replicate containing eight hens, for a total of 37 days trial (including a 7-day adaptation period). The treatments included a control group (0% SFL in drinking water) and drinking water supplemented with 0.3, 0.5, or 0.7% SFL.

**Results:**

The fermentation optimization conditions for Shuanghuanglian were selected as a solid-to-liquid ratio at 1:7, 3% inoculation quantity, fermentation temperature at 28°C for 5 days, initial pH of 7, 60 mesh (sieved), and rotation speed of 150 r/min. Various bioactive compounds, such as myrtenol, 2-hexyn-1-ol, arsenous acid tris(trimethylsilyl) ester, 3(10)-caren-4-ol, and oxime-, methoxy-phenyl, were detected in SFL. The most abundant bacterial phyla in SFL were Proteobacteria and Firmicutes, with *Acinetobacter* being the most abundant genus. The most abundant fungal phyla were Phragmoplastophyta and *Magnoliophyta*. The 0.5 and 0.7% SFL supplementation in water increased egg weight and laying rate, while decreasing the feed-to-egg ratio of laying hens compared with the control group (*p* < 0.05). Additionally, 0.3, 0.5, and 0.7% SFL supplementation in water increased (*p* < 0.05) the Haugh unit, but there were no significant differences (*p* > 0.05) in albumen height, egg shape index, egg thickness, and yolk color of the eggs.

**Conclusion:**

Supplementation of SFL under optimized conditions had a positive impact on the production performance of laying hens, especially when the supplementation amount reached 0.5%. This study provides a theoretical basis for the application of Shuanghuanglian in the commercial egg industry.

## 1 Introduction

Shuanghuanglian is a Chinese medicine that consists of Chinese natural herbs, including *Honeysuckle Flower*, *Baical Skullcap Root*, and *Fructus Forsythiae* in a 1:1:2 ratio (1). It has been found to have various biological functions, such as antipyretic, anti-inflammatory, antibacterial, antiviral, and immune-enhancing effects, and is widely used for respiratory tract infections, mild pneumonia, and common cold treatments caused by virus and bacterial infection (2, 3). Recent studies have also shown that Shuanghuanglian can regulate the structure and function of the intestinal microbiota in rats (3), indicating that it may have potential to improve the intestinal health of poultry and livestock and therefore influence their production performance. Although Shuanghuanglian is widely used as a safe drug in human medicine (4), there is limited information available regarding its use in animal husbandry.

A previous study found that supplementing drinking water with Shuanghuanglian liquid improved the immune performance of broilers (5). Therefore, we hypothesized that Shuanghuanglian might have positive effects on the laying performance and egg quality of laying hens by reducing diseases. Since Shuanghuanglian is commonly used as an oral liquid (2–5) and the cell walls of Chinese herbal medicines typically limit the release of bioactive components (6), we used lignin-degrading bacteria (*Bacillus amyloliquefaciens-c4*) to ferment Shuanghuanglian to release the bioactive components into Shuanghuanglian fermentation liquor (SFL). Consequently, we optimized the fermentation conditions of Shuanghuanglian using single factor and response surface methodology, evaluated the compound and microbial composition of SFL, and further supplemented SFL in drinking water for laying hens. Albumen height, egg shape index, eggshell thickness, Haugh unit, and yolk color were determined as important indexes of egg quality (7, 8). This study will provide a theoretical basis for the application of Shuanghuanglian in laying hens.

## 2 Materials and methods

### 2.1 Sources of Shuanghuanglian and microbial inoculum

*Honeysuckle Flower*, *Baical Skullcap Root*, and *Fructus Forsythiae* were provided by Fuyang Normal University, Fuyang, Anhui, China. The microbial inoculum (*Bacillus amyloliquefaciens-c4*; preservation number CGMCC No. 15178) was provided by the Biological Inoculation Research and Development Center of Northeast Agricultural University, Harbin, Heilongjiang, China.

### 2.2 Optimization of microbial fermentation conditions by single factor experiment

Shuanghuanglian [not sieved; solid-to-liquid ratio (SLR) of 1:5] was added with 5% brown sugar. It was then inoculated with 5% microbial inoculum and fermented at 28°C for 7 days using a constant temperature shaker (Shidan Equipment Inc., Shanghai, China) with a rotational speed of 180 r/min.

The following conditions were used to determine fermentation optimization were as follows: (1) the SLR was adjusted to 1:1, 1:3, 1:5, 1:7, and 1:9, while keeping other fermentation conditions unchanged, (2) the inoculation quantity of microbial inoculum was adjusted to 1, 3, 5, 7, and 9%, while keeping other conditions unchanged, (3) the fermentation temperature was set to 24°C, 28°C, 33°C, 37°C, and 42°C using the constant temperature shaker (Shidan Equipment Inc., Shanghai, China), while keeping other conditions unchanged, (4) the fermentation time was set to 1, 3, 5, 7, and 9 days, while keeping other conditions unchanged, (5) the initial pH value was adjusted to 5, 6, 7, 8, and 9 using a pH meter (Gaozhi Precision Instrument Inc., Shanghai, China), while keeping other conditions unchanged, (6) the particle size was set to dp (decoction pieces form without sieving), 20, 40, 60, and 80 mesh using sieves (ZhenXing Inc., Guangzhou, China), while keeping other conditions unchanged, and (7) the rotational speed was set to 0, 120, 150, 180, and 210 r/min using the constant temperature shaker (Shidan Equipment Inc., Shanghai, China), while keeping other conditions unchanged. Each fermentation condition was replicated three times. Finally, the surface morphology of the three component medicines, chemical and microbial compositions, and feeding effects of Shuanghuanglian on laying hens were evaluated based on the optimized fermentation conditions.

### 2.3 Chemical compound composition analysis

The dry matter content of the Shuanghuanglian was analyzed using previously described methods by Esfahani and Goli (9). Chlorogenic acid, baicalin, and phillyrin were extracted using the ChP method (1). Briefly, 1 mL of SFL was added to 5 mL of 50% (*w*/*v*) methanol and sonicated for 25 min using a multifunctional constant-temperature ultrasonic extractor (Scientz-1000C, Xinzhi Biotechnology Inc., Ningbo, China) to obtain the supernatant for analysis. The chlorogenic acid, baicalin, and phillyrin contents were measured as previously described by Li et al. (10) using an ultraviolet spectrophotometer (T6 New Century, Puxi General Instrument Inc., Beijing, China). Additionally, the supernatants obtained from sonication were concentrated 100 times using a rotary evaporator (Hei-VAP Advantage ML/HB/G3, Heidolph, Schwabach, Germany) and filtered through a 0.22 μm filter membrane to determine the chemical composition of the SFL using a gas chromatography–mass spectrometer (Agilent 7890A/5975C, Agilent, Santa Clara, United States). The chemical composition of the SFL was analyzed in triplicate.

### 2.4 Observation of surface morphology

The surface morphologies of *Honeysuckle Flower*, *Baical Skullcap Root*, and *Fructus Forsythiae* were examined using a field emission scanning electron microscopy (FESEM; model SU8010; Hitachi, Japan), as previously described by Yi et al. (11). Images representing the average characteristics of the surface morphology were screened at a magnification of 1,000×.

### 2.5 Analysis of microbiota composition

The composition of the microbiota was analyzed following the methods previously described by Yi et al. (11). Briefly, the total microbial DNA of the SFL was extracted using the TIANamp Stool DNA Kit (Tiangen Biochemical Technology Inc., Beijing, China). The concentration and purity of the extracted DNA were determined using a NanoDrop ND-2000 spectrophotometer (Thermo Fisher Inc., Waltham, MA, United States). The V3–V4 region of the 16S rRNA genes was amplified using the bacterial universal primers 341F (5′-ACTCCTACGGGAGGCAGCAG-3′) and 806R (5′-GGACTACHVGGGTWTCTAAT-3′). Similarly, the V4 region of the 18S rRNA genes was amplified using the fungal universal primers F (5′-CCAGCASCYGCGGTAATTCC-3′) and R (5′-ACTTTCGTTCTTGATYRA-3′). The sequencing of the 16S rRNA and 18S rRNA was performed using the Illumina NovaSeq platform (Illumina Inc., San Diego, CA, United States) by Boyuezhihe Biology Science and Technology Co., Ltd. (Wuhan, China). The analysis of the microbiota composition of the SFL was performed in triplicate.

### 2.6 Animals, treatment, and feeding

The animal trial was conducted at Duoduoli Agricultural Science and Technology Co., Ltd., Fuyang, China. A total of 288 healthy Xinyang black laying hens, 50 week-old (laying rate < 80%) and with similar body weight, were selected for the feeding trial. After a 7-days adaptation period, the laying hens were divided into four groups using a completely randomized block design. Each group was fed with different SFL levels in water: 0 (control), 0.3, 0.5, and 0.7%. Each group consisted of nine replicates with eight hens per replicate. The composition and nutrient levels of the basal diets are presented in Table 1. The experimental laying hens were reared at the Ancient West Lake Modern Agricultural Science and Technology Demonstration Park in Fuyang, China, and were fed twice daily at 06:00 and 18:00. All the hens had free access to water at all times. The SFL was mixed with drinking water before feeding. The experiment lasted for 37 days, including the adaptation period.

**Table 1:** Ingredients and chemical compositions of basal diet (as DM basis).

Item	Value
Ingredient, g/kg	
Corn grain	645.08
Soybean meal	225.50
Limestone powder	88.90
DL-methionine	0.52
Premix^a^	40.00
Nutrient levels^b^, g/kg	
Available phosphorus	3.30
Calcium	32.30
Crude protein	168.0
Metabolizable energy, MJ/kg	11.54
Lysine	6.70
Methionine	3.10
Total phosphorus	4.60

a Providing the following amounts of vitamins and minerals per kg of a complete diet: vitamin A, 10, 000 IU; vitamin D_3_, 2, 500 IU; vitamin E, 18 IU; vitamin K_3_, 1 mg; vitamin B_1_, 2 mg; vitamin B_2_, 6 mg; vitamin B_6_, 3.5 mg; vitamin B_12_, 15 μg; nicotinic acid, 63 mg; pantothenic acid, 18 mg; folic acid, 0.4 mg; biotin 0.15 mg; Fe, 80 mg; Cu, 9 mg; Zn, 70 mg; Mn, 80 mg; I, 0.6 mg; and Se, 0.3 mg.

b The data of crude protein was analyzed in triplicate according to AOAC methods No. 990.03, the calcium and total phosphorus were analyzed in triplicate according to AOAC methods No. 968.08 (12). Available phosphorus, metabolizable energy, lysine, and methionine levels were calculated.

### 2.7 Determination of laying performance and egg quality

The egg production, egg weight, feed offered, and refusals were recorded daily to calculate average egg weight, average daily feed intake (ADFI), feed-to-egg ratio, and laying rate.

Eggshell thickness without egg membrane was measured as the mean of the thickness at either end and in the middle of the shell using a screw micrometer (211-115, Sanliang Measuring Tool Inc., Dongguan, China) to calculate the eggshell thickness. The albumin height and yolk color were measured with a multifunctional egg quality meter (EA-01, Tenovo International Inc., Beijing, China). The egg shape index was calculated from the longitudinal and transverse diameters of the eggs using Vernier calipers (171-502, Sanliang Measuring Tool Inc., Dongguan, China). The Haugh units of eggs were calculated using the albumen height and egg weight.

### 2.8 Response surface experimental design and statistical analysis

The response surface experiments were designed and analyzed using Design-Expert 13.0 software, following the methods described by Mohammadabadi et al. (13). The independent variables were the initial pH and temperature, initial pH and rotational speed, and temperature and rotational speed. The initial pH, temperature, and rotational speed ranges were 6–7, 24–32°C, and 120–180 r/min, respectively. The dependent variables included chlorogenic acid, baicalin, and phillyrin. Regression models for these variables were constructed based on the data obtained in this study. The accuracy of the regression models was assessed using *R*^2^, *R*^adj^, C.V.%, and Adep precision values.

The data for the optimized fermentation conditions and the production performance of laying hens were analyzed using one-way ANOVA, and the comparative analysis among different treatment groups was analyzed by Duncan’s multiple range test with SPSS software (version 26.0; SPSS Inc., Chicago, IL, United States). The data for the optimized fermentation conditions are presented as means and standard errors, and the data for the production performance of hens are presented as means and standard errors of the mean (SEM). Statistical significance was set at *p* < 0.05.

## 3 Results

### 3.1 Optimization of microbial fermentation conditions of Shuanghuanglian by single factor experiments

**Figure 1:**
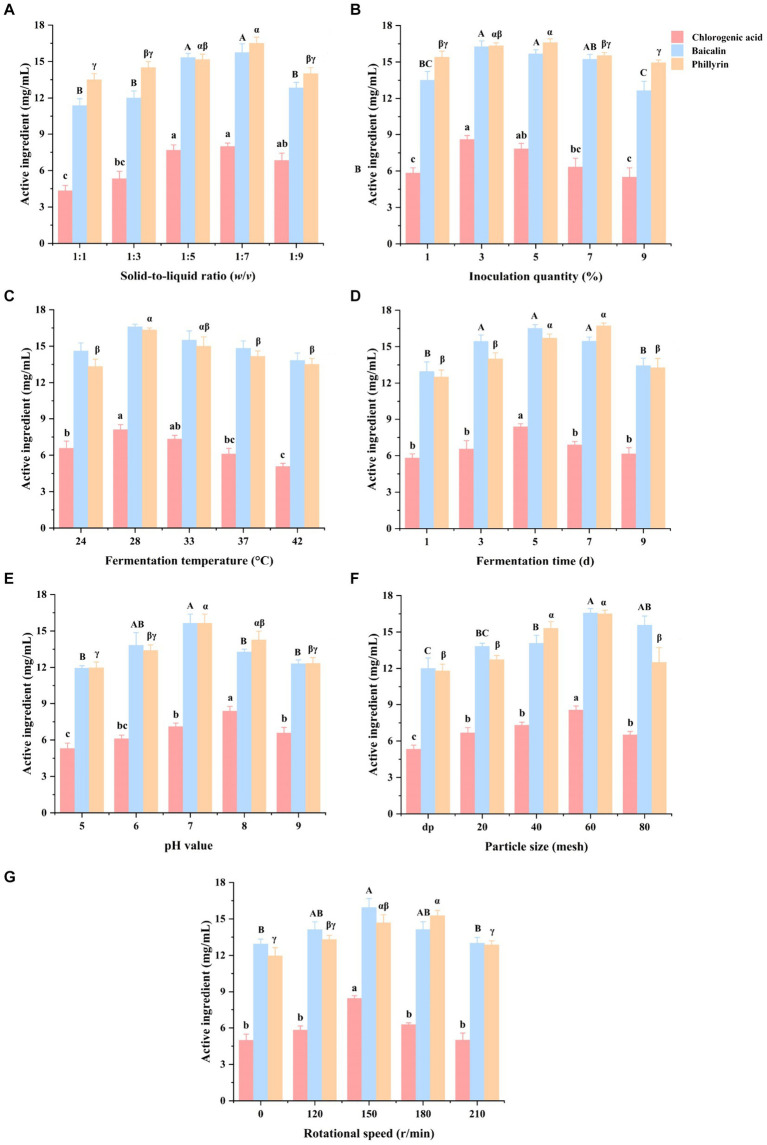
Effects of solid-to-liquid ratio **(A)**, inoculation quantity **(B)**, temperature **(C)**, time **(D)**, initial pH **(E)**, particle size **(F)**, and rotational speed **(G)** on chlorogenic acid, baicalin, and phillyrin concentrations of Shuanghuanglian fermentation liquor (*n* = 3). d, days; dp, decoction pieces form without sieving; data are presented as means ± standard error. Different letters in the same index indicate significant differences (*p* < 0.05).

The results of the optimized fermentation conditions of Shuanghuanglian in a single-factor experiment are shown in Figure 1. Compared with the 1:5 and 1:7 SLR, Shuanghuanglian fermented at 1:1 and 1:3 SLR had a lower (*p* < 0.05) chlorogenic acid concentration, while Shuanghuanglian fermented at 1:1, 1:3, and 1:9 SLR had a lower (*p* < 0.05) baicalin concentration. Furthermore, Shuanghuanglian fermented at 1:7 SLR had a higher (*p* < 0.05) phillyrin concentration than the 1:1, 1:3, or 1:9 SLR (Figure 1A).

In term of inoculation quantities, Shuanghuanglian fermented with 3% inoculation had a higher (*p* < 0.05) chlorogenic acid concentration than the 1, 7, and 9% inoculation. Shuanghuanglian fermented with 3 and 5% inoculation quantities had a higher (*p* < 0.05) baicalin concentration compared to the 1 and 9% inoculation. Moreover, Shuanghuanglian fermented with 5% inoculation had a higher (*p* < 0.05) phillyrin concentration than the 1, 7, and 9% inoculation (Figure 1B).

Compared with the fermentation temperatures at 24°C, 37°C, and 42°C, Shuanghuanglian fermented at 28°C had higher (*p* < 0.05) concentrations of chlorogenic acid and phillyrin. However, there was no significant difference (*p* > 0.05) in baicalin concentration among the different fermentation temperatures (including 24°C, 28°C, 33°C, 37°C, and 42°C) (Figure 1C).

Among the different fermentation times, Shuanghuanglian fermented for 5 days had a higher (*p* < 0.05) chlorogenic acid concentration compared to the other fermentation times (1, 3, 7, and 9 days). Shuanghuanglian fermented for 3, 5, and 7 days had a higher (*p* < 0.05) baicalin concentration compared to those fermented for 1 or 9 days. Furthermore, Shuanghuanglian fermented for 5 and 7 days had a higher (*p* < 0.05) phillyrin concentration than the 1, 3, and 9 days (Figure 1D).

When considering the initial pH values, Shuanghuanglian fermented with an initial pH of 8 had a higher (*p* < 0.05) chlorogenic acid concentration compared to the other initial pH values (5, 6, 7, and 9). Shuanghuanglian fermented with an initial pH of 7 had a higher (*p* < 0.05) baicalin concentration than the pH values of 5, 8, or 9. Moreover, Shuanghuanglian fermented with an initial pH value of 7 had a higher (*p* < 0.05) phillyrin concentration than the initial pH values of 5, 6, or 9 (Figure 1E).

Among the different particle sizes, Shuanghuanglian fermented with a particle size of 60 mesh had a higher (*p* < 0.05) chlorogenic acid concentration than other particle sizes (dp, 20, 40, and 80 mesh). Furthermore, Shuanghuanglian fermented with a particle size of 60 mesh had a higher (*p* < 0.05) baicalin concentration than the dp, 20, and 40 mesh, while Shuanghuanglian fermented with 40 and 60 mesh particle sizes had a higher (*p* < 0.05) phillyrin concentration compared to dp, 20, and 80 mesh (Figure 1F).

Compared with the 150 r/min, Shuanghuanglian fermented at 0, 120, 180, and 210 r/min had a lower (*p* < 0.05) chlorogenic acid concentration. Shuanghuanglian fermented at 150 r/min had a higher (*p* < 0.05) baicalin concentration than at the 0 and 210 r/min. Furthermore, Shuanghuanglian fermented at a rotational speed of 180 r/min had a higher (*p* < 0.05) phillyrin concentration than the 0, 120, and 210 r/min (Figure 1G).

### 3.2 Variance analysis of response surface model

Based on the single-factor experiments, three factors (initial pH, temperature, and rotational speed) were considered to verify the response surface experiments with significant differences. The design and results of the optimized fermentation of Shuanghuanglian by the response surface experiments are shown in Tables 2, 3.

**Table 2:** Factors and levels of response surface experiment.

Factors	Levels		
	−1	0	1
pH	6	7	8
Temperature (°C)	24	28	32
Rotational speed (r/min)	120	150	180

**Table 3:** Design and results of response surface experiment.

Design				Results		
Serial number	initial pH	Temperature (°C)	Rotational speed (r/min)	Chlorogenic acid (mg/mL)	Baicalin (mg/mL)	Phillyrin (mg/mL)
1	0	1	1	6.58	14.48	12.75
2	1	−1	0	6.11	11.46	14.08
3	1	0	0	8.38	16.89	17.54
4	0	1	−1	6.14	13.32	13.18
5	−1	0	1	6.39	13.77	12.80
6	1	0	1	5.83	11.55	11.93
7	0	0	0	8.37	17.39	16.95
8	1	1	0	5.65	13.20	12.45
9	0	0	0	8.26	17.59	16.5
10	0	0	0	8.26	17.10	17.35
11	0	0	0	8.36	17.05	17.17
12	0	−1	1	6.75	11.71	14.39
13	0	−1	−1	6.95	11.32	15.59
14	−1	0	−1	6.03	11.23	12.06
15	−1	1	0	5.89	14.32	12.21
16	1	0	−1	6.01	12.84	14.31
17	−1	−1	0	6.44	11.25	13.24

The variance analysis of chlorogenic acid is presented in Table 4. The regression model for chlorogenic acid is *Y* = 8.33 − 0.14A − 0.25B + 0.053C + 0.023AB − 0.14 AC + 0.16 BC − 1.42A^2^ − 0.88B^2^ − 0.84C^2^. Where *Y* represents the chlorogenic acid concentration (mg/mL), and A, B, and C represent initial pH, temperature (°C), and rotational speed (r/min), respectively. There was a significant difference (*p* < 0.05) in the regression model and no difference (*p* > 0.05) in the lack-of-fit. The factors A, B, C, AC, BC, A^2^, B^2^, and C^2^ had significant differences (*p* < 0.05), indicating that they had a significant influence on the chlorogenic acid concentration. The *R*^2^ and *R*^adj^ values of the regression model were 0.999 and 0.998, respectively. The C.V.% and Adep Precision were 0.70 and 72.39, respectively, indicating that the model has high accuracy and credibility.

**Table 4:** Variance analysis of response surface model for chlorogenic acid.

Item	Sum of variance	Freedom	Mean square	*F*-values	*p*-values
Model	17.23	9	1.91	824.24	<0.001
A	0.17	1	0.17	71.19	<0.001
B	0.50	1	0.50	213.17	<0.001
C	0.02	1	0.02	9.50	0.018
AB	2.03 × 10^−4^	1	2.03 × 10^−3^	0.87	0.382
AC	0.07	1	0.07	31.39	0.001
BC	0.10	1	0.10	44.10	<0.001
A^2^	8.52	1	8.52	3670.33	<0.001
B^2^	3.28	1	3.28	1412.94	<0.001
C^2^	2.97	1	2.97	1280.16	<0.001
Residual	0.02	7	2.32 × 10^−3^		
Lack of fit	5.75 × 10^−4^	3	1.92 × 10^−4^	0.05	0.984
Error term	0.02	4	3.92 × 10^−3^		
Summation	17.24	16	1.91		

A, initial pH; B, temperature; C, rotational speed.

The variance analysis of baicalin is shown in Table 5. The regression model of baicalin is *Y* = 17.20 − 0.19A + 1.20B + 0.35C − 0.33AB − 0.96 AC + 0.19 BC − 2.50A^2^ − 2.14B^2^ − 2.35C^2^. Where *Y* represents the baicalin concentration (mg/mL), and A, B, and C represent initial pH, temperature (°C), and rotational speed (r/min), respectively. There was a significant difference (*p* < 0.05) in the regression model and no difference (*p* > 0.05) in the lack-of-fit. The factors A, B, C, AB, AC, A^2^, B^2^, and C^2^ had significant differences (*p* < 0.05), indicating that they had a great effect on the baicalin concentration. The *R*^2^ and *R*^adj^ values of the regression model were 0.996 and 0.992, respectively. The C.V.% and Adep Precision were 1.58 and 35.45, respectively, indicating that the model has high accuracy and credibility.

**Table 5:** Variance analysis of response surface model for baicalin.

Item	Sum of variance	Freedom	Mean square	*F*-values	*p*-values
Model	94.12	9	10.46	215.67	<0.001
A	0.29	1	0.29	5.96	0.045
B	11.47	1	11.47	236.59	<0.001
C	0.98	1	0.98	20.21	0.003
AB	0.44	1	0.44	9.12	0.019
AC	3.67	1	3.67	75.63	<0.001
BC	0.15	1	0.15	3.06	0.124
A^2^	26.38	1	26.38	544.13	<0.001
B^2^	19.34	1	19.34	398.88	<0.001
C^2^	23.32	1	23.32	480.88	<0.001
Residual	0.32	4	0.08		
Lack of fit	0.34	7	0.05		
Error term	0.02	3	7.57 × 10^−3^	0.10	0.959
Summation	94.46	16			

A, initial pH; B, temperature; C, rotational speed.

The variance analysis of phillyrin is presented in Table 6. The regression model for phillyrin is *Y* = 17.10 + 0.31A − 0.84B − 0.41C − 0.15AB − 0.78 AC + 0.19 BC − 2.65A^2^ − 1.45B^2^ − 1.67C^2^. Where *Y* represents the phillyrin concentration (mg/mL), and A, B, and C represent initial pH, temperature (°C), and rotational speed (r/min), respectively. There was a significant difference (*p* < 0.05) in the model and no difference (*p* > 0.05) in the lack-of-fit. The factors A, B, C, AC, A^2^, B^2^, and C^2^ had significant differences (*p* < 0.05), indicating that they had great effects on the phillyrin concentration. The *R*^2^ and *R*^adj^ values of the regression model were 0.987 and 0.969, respectively. The C.V.% and Adep Precision were 2.49 and 18.98, respectively, indicating that the model has high accuracy and credibility.

**Table 6:** Variance analysis of response surface model for phillyrin.

Item	Sum of variance	Freedom	Mean square	*F*-values	*p*-values
Model	66.06	9	7.34	57.33	<0.001
A	0.76	1	0.76	5.91	0.045
B	5.63	1	5.63	43.96	<0.001
C	1.34	1	1.34	10.44	0.014
AB	0.09	1	0.09	0.70	0.430
AC	2.43	1	2.43	19.01	0.003
BC	0.15	1	0.15	1.16	0.318
A^2^	29.67	1	29.67	231.77	<0.001
B^2^	8.88	1	8.88	69.36	<0.001
C^2^	11.77	1	11.77	91.96	<0.001
Residual	0.90	7	0.13		
Lack of fit	0.25	3	0.08	0.52	0.689
Error term	0.64	4	0.16		
Summation	66.96	16			

A, initial pH; B, temperature; C, rotational speed.

### 3.3 Optimization of microbial fermentation conditions of Shuanghuanglian by response surface experiment

**Figure 2:**
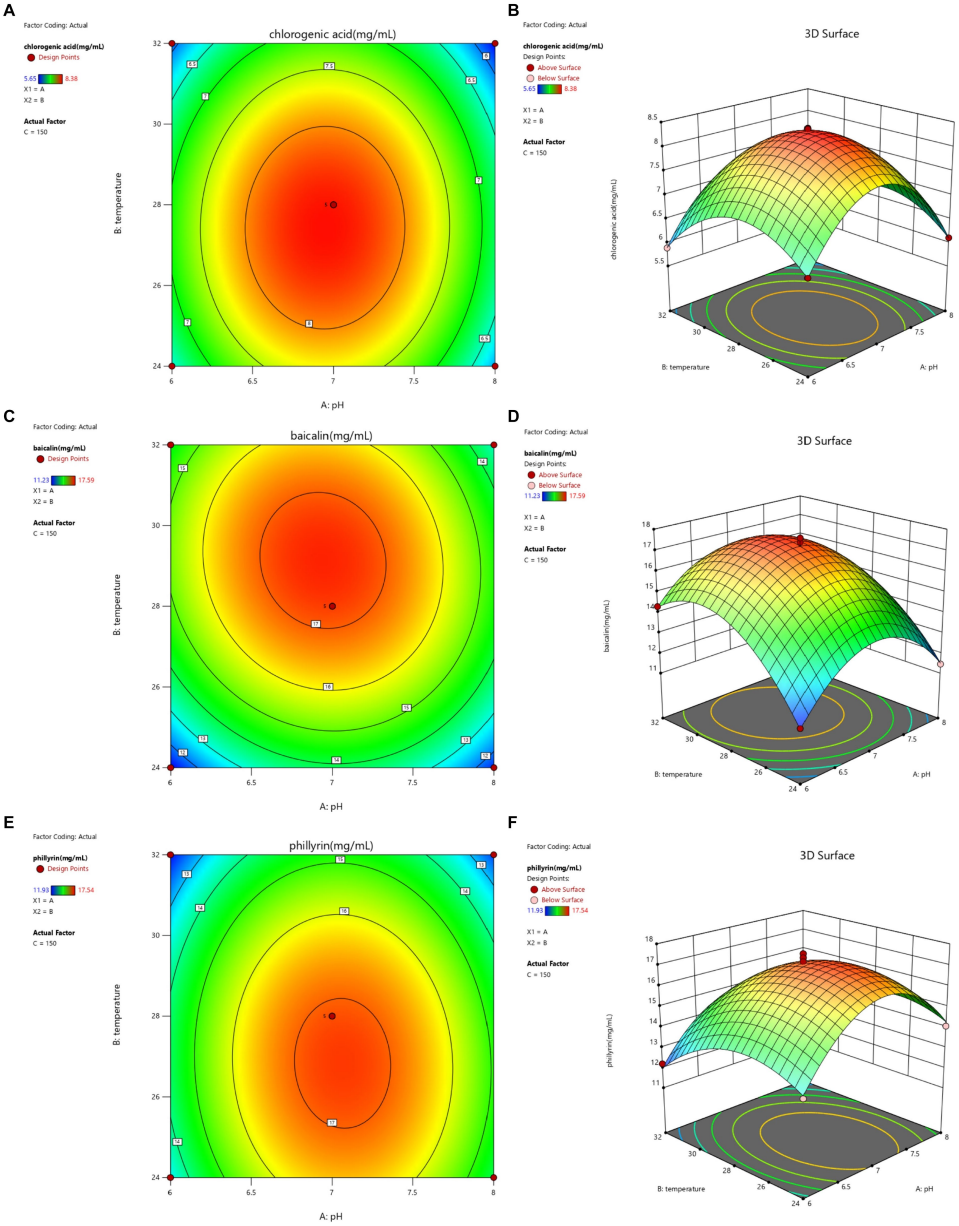
Contour lines and response surface of initial pH and temperature to chlorogenic acid **(A,B)**, baicalin **(C,D)**, and phillyrin **(E,F)** concentrations of Shuanghuanglian fermentation liquor.

**Figure 3:**
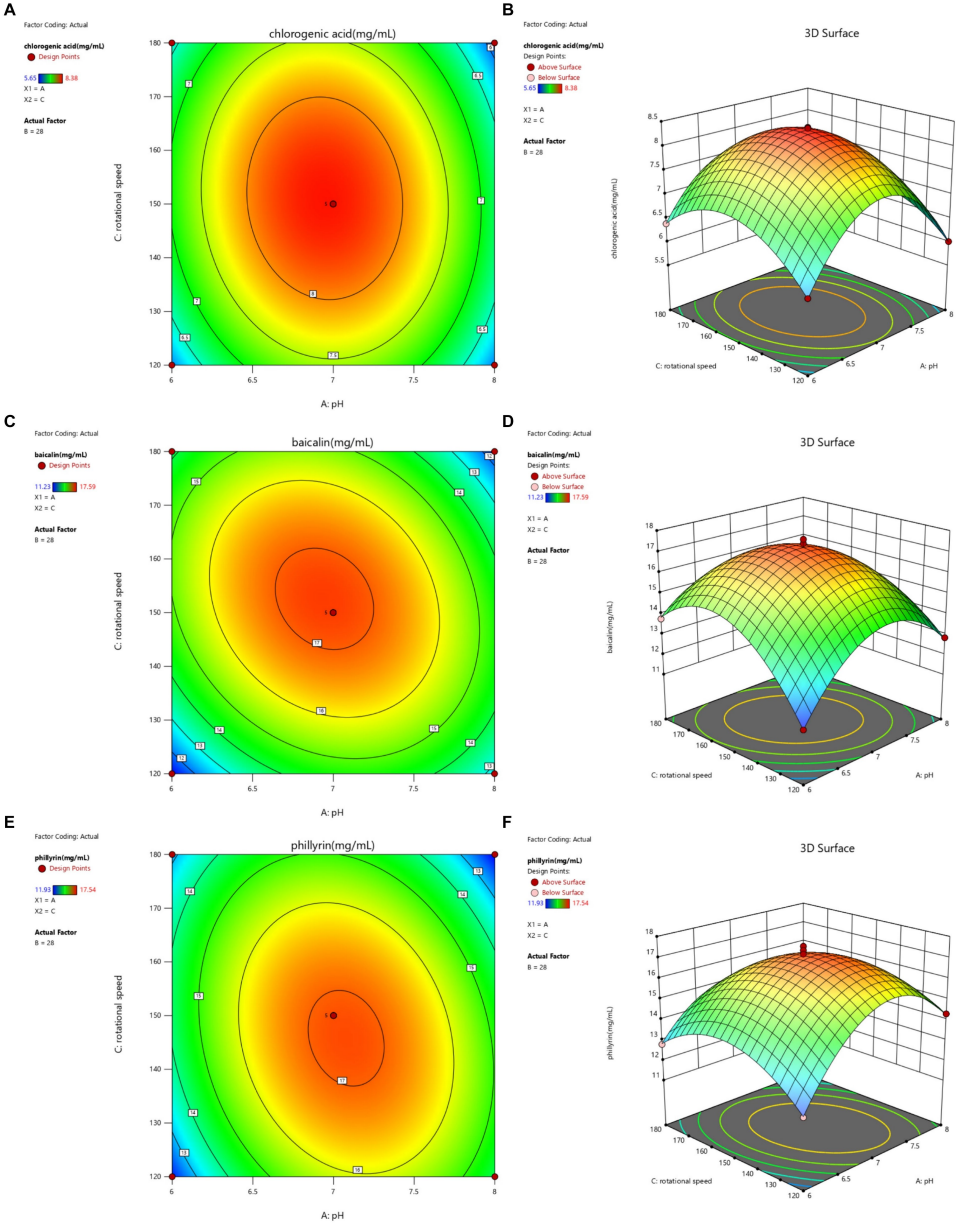
Contour lines and response surface of initial pH and rotational speed to chlorogenic acid **(A,B)**, baicalin **(C,D)**, and phillyrin **(E,F)** concentrations of Shuanghuanglian fermentation liquor.

**Figure 4:**
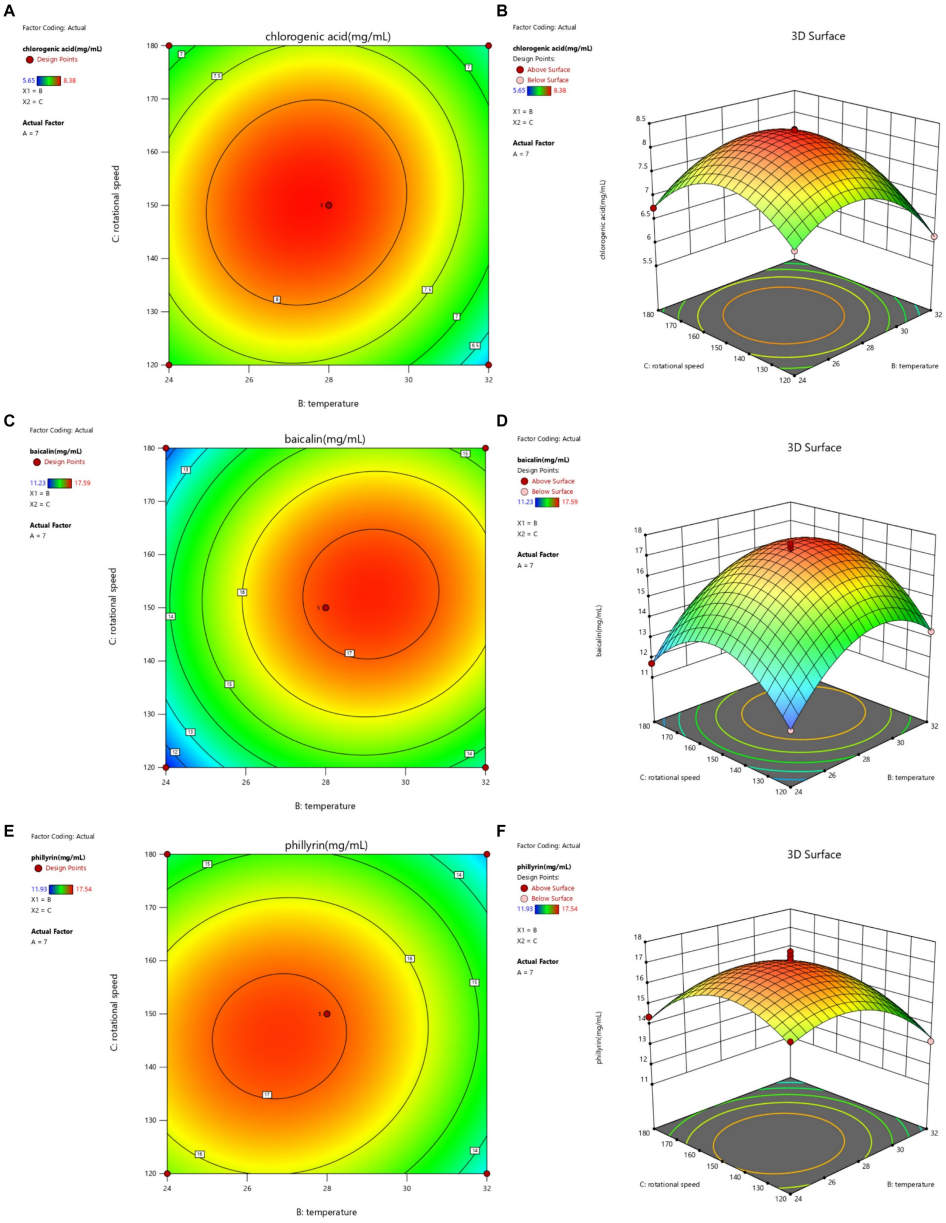
Contour lines and response surface of temperature and rotational speed to chlorogenic acid **(A,B)**, baicalin **(C,D)**, and phillyrin **(E,F)** concentrations of Shuanghuanglian fermentation liquor.

The contour lines and response surface of the optimized fermentation of Shuanghuanglian are shown in Figures 2–4. The effects of the interaction between initial pH and temperature on the concentrations of chlorogenic acid, baicalin, and phillyrin in SFL are shown in Figure 2. The contour lines for chlorogenic acid were circular, indicating that the interaction between pH and temperature had no significant effect on chlorogenic acid. However, the concentration of chlorogenic acid reached its highest point, and pH and temperature had significant effects on the concentration of chlorogenic acid. The contour lines for baicalin and phillyrin were elliptical, and the interaction between pH and temperature significantly affected baicalin and phillyrin. Temperature and pH had more significant effects on the concentration of baicalin than the phillyrin. Compared with the effects of pH on baicalin and phillyrin concentrations, temperature had greater effects, and there was also a significant interaction between pH and temperature. When the pH value was fixed, the concentrations of chlorogenic acid, baicalin, and phillyrin increased initially and then decreased with increasing temperature. At the same temperature, the concentrations of the three bioactive components increased initially and then decreased, and the concentrations reached a maximum with increasing pH value. Overall, the highest concentrations of chlorogenic acid, baicalin, and phillyrin were observed within the temperature range of 27–30°C and the pH value of 6.5–7.5.

The interactions between initial pH and rotational speed on the chlorogenic acid, baicalin, and phillyrin concentrations in the SFL are shown in Figure 3. The contour lines for chlorogenic acid, baicalin, and phillyrin observed as oval shapes, and the interaction between pH and rotational speed had significant effects on the chlorogenic acid, baicalin, and phillyrin concentrations. Compared to the rotational speed on chlorogenic acid concentration, the pH value had a greater effect on its concentration. Compared with the effects of pH on baicalin and phillyrin concentrations, the effects of rotational speed on their concentrations were more significant. When the pH value was constant, the chlorogenic acid, baicalin, and phillyrin concentrations displayed an increasing trend initially and then decreased with increasing rotating speed, whereas when the rotational speed was constant, the three bioactive components displayed an increasing trend initially and then decreased with increasing pH value. Overall, the highest concentrations of chlorogenic acid, baicalin, and phillyrin were observed at a rotational speed of 145–155 r/min and the pH value of 6.5–7.5.

The interactions between temperature and rotational speed on chlorogenic acid, baicalin, and phillyrin concentrations in SFL are shown in Figure 4. The contours lines for baicalin and phillyrin concentrations were circular, and the interaction between temperature and rotational speed had no significant effects on baicalin and phillyrin concentrations. However, baicalin and phillyrin reached their highest concentration points, and the rotational speed and temperature had significant effects on baicalin and phillyrin concentrations. Temperature had a more significant effects on baicalin and phillyrin concentrations than the rotational speed. The contour line for chlorogenic acid concentration was elliptical, and the interaction between temperature and rotational speed had significant effects on the chlorogenic acid concentration. Temperature had a more significant effect on chlorogenic acid concentration than the rotational speed. When the temperature was constant, the chlorogenic acid, baicalin, and phillyrin concentrations displayed an increasing trend initially and then decreased with an increasing rotational speed. At a constant rotational speed, the three bioactive components initially increased and then decreased with the increasing temperature. Overall, the highest concentrations of chlorogenic acid, baicalin, and phillyrin appeared with a rotational speed of 145–155 r/min and temperature of 27–30°C.

The optimized fermentation conditions were determined as follows: an initial pH of 6.99, temperature of 27.89°C, and rotational speed of 149.99 r/min. Considering the application of production, the fermentation conditions were set with an initial pH of 7, temperature at 28°C, and rotational speed of 150 r/min. Under these conditions, the concentrations of chlorogenic acid, baicalin, and phillyrin were 9.36, 18.52, and 17.63 mg/mL, respectively. These concentrations were not significantly different from those predicted by the response surface model (concentrations of chlorogenic acid, baicalin, and phillyrin were 8.33, 17.17, and 17.12 mg/mL, respectively). Compared to Shuanghuanglian fermented under basic conditions, the concentrations of chlorogenic acid, baicalin, and phillyrin were increased by 75.60, 43.23, and 47.40%, respectively, indicating that the optimized technological parameters were accurate and reliable, and the response surface model could accurately predict the concentrations of chlorogenic acid, baicalin, and phillyrin in SFL.

Finally, the optimized fermentation conditions for Shuanghuanglian were selected as a 1:7 SLR, 3% inoculation quantity, 28°C fermentation for 5 days, initial pH of 7, 60 mesh particle size, and a rotational speed of 150 r/min.

### 3.4 Chemical composition of Shuanghuanglian fermentation liquor

The chemical composition of SFL is presented in Table 7. Compounds with relative proportions ≤1.0% and qualitative values ≤50% were excluded from further analysis. The baicalin, phillyrin, and chlorogenic acid concentrations in SFL were 18.52, 17.63, and 9.36 mg/mL, respectively. Furthermore, (2E,5E)-2,5-heptadiene had the highest proportion (4.80%) in SFL, followed by myrtenol (4.45%), 2,6-di-tert-butylnaphthalene (3.25%), decamethylcyclopentasiloxane (2.60%), 2-hexyn-1-ol (2.11%), arsenous acid tris(trimethylsilyl) ester (2.04%), 3(10)-caren-4-ol (1.81%), decamethyltetrasiloxane (1.67%), 3-hydroxymethylbenzamide (1.39%), 2-isopropylidenecycloheptanone semicarbazone (1.19%), and oxime-, methoxy-phenyl (1.09%).

**Table 7:** Compound compositions (% of total compounds) of Shuanghuanglian fermentation liquor.

Item	Value
(2E,5E)-2,5-Heptadiene	4.80
Myrtenol	4.45
2,6-Di-tert-butylnaphthalene	3.25
Decamethylcyclopentasiloxane	2.60
2-Hexyn-1-ol	2.11
Arsenous acid tris(trimethylsilyl) ester	2.04
3(10)-Caren-4-ol	1.81
Decamethyltetrasiloxane	1.67
3-Hydroxymethylbenzamide	1.39
2-Isopropylidenecycloheptanone semicarbazone	1.19
Oxime-, methoxy-phenyl	1.09
Others	73.62

### 3.5 The surface morphology of *honeysuckle flower*, *Baical skullcap root*, and *Fructus Forsythiae*

**Figure 5:**
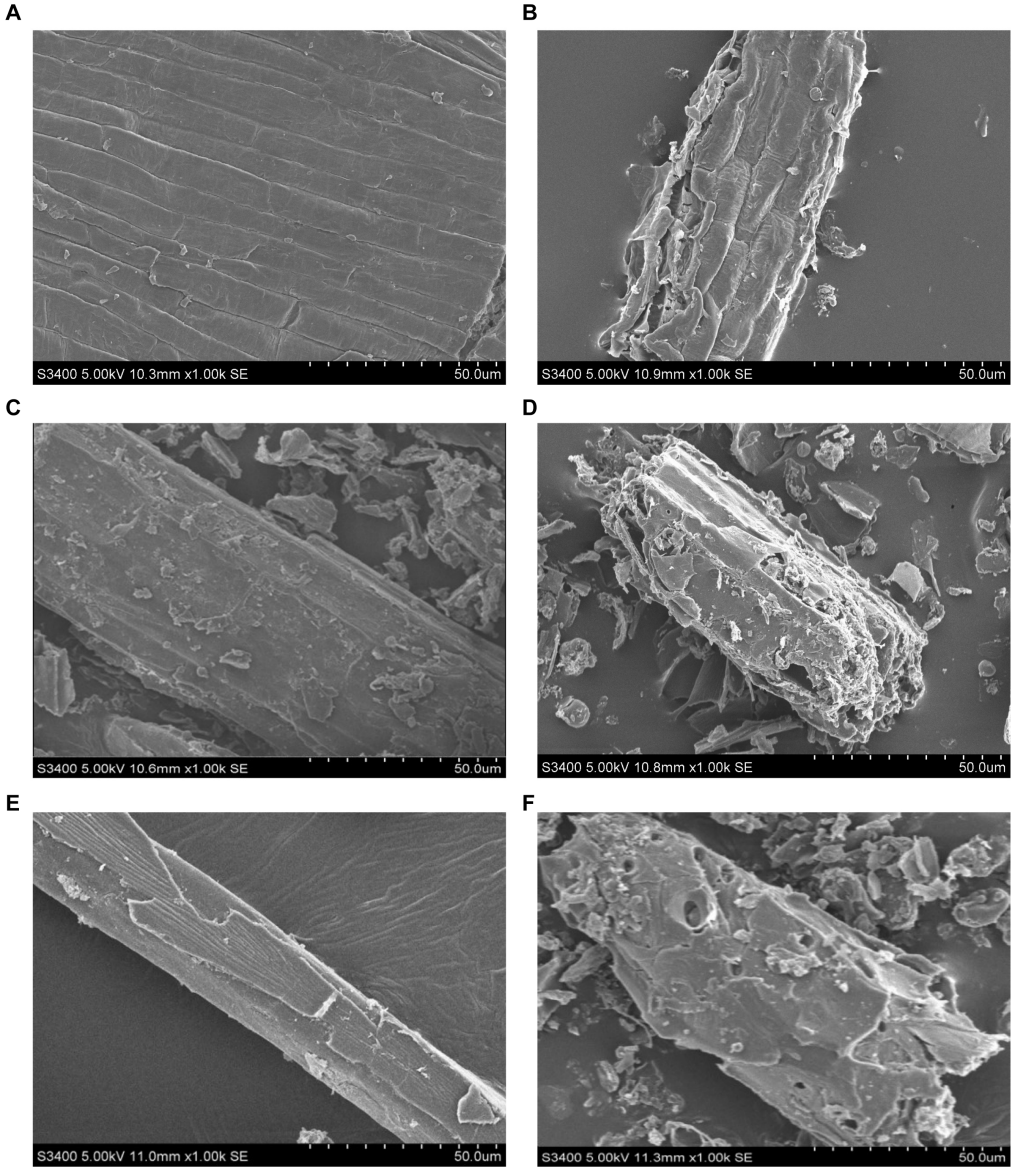
Scanning electron microscopy images of *Honeysuckle Flower* **(A,B)**, *Baical Skullcap Root* **(C,D)**, and *Fructus Forsythiae* **(E,F)** before and after fermentation.

Scanning electron microscopy analyses showed that the cell walls of *Honeysuckle Flower* (Figures 5A,B), *Baical Skullcap Root* (Figures 5C,D), and *Fructus Forsythiae* (Figures 5E,F) were more broken after fermentation compared to before fermentation.

### 3.6 Microbiota composition of Shuanghuanglian fermentation liquor

**Figure 6:**
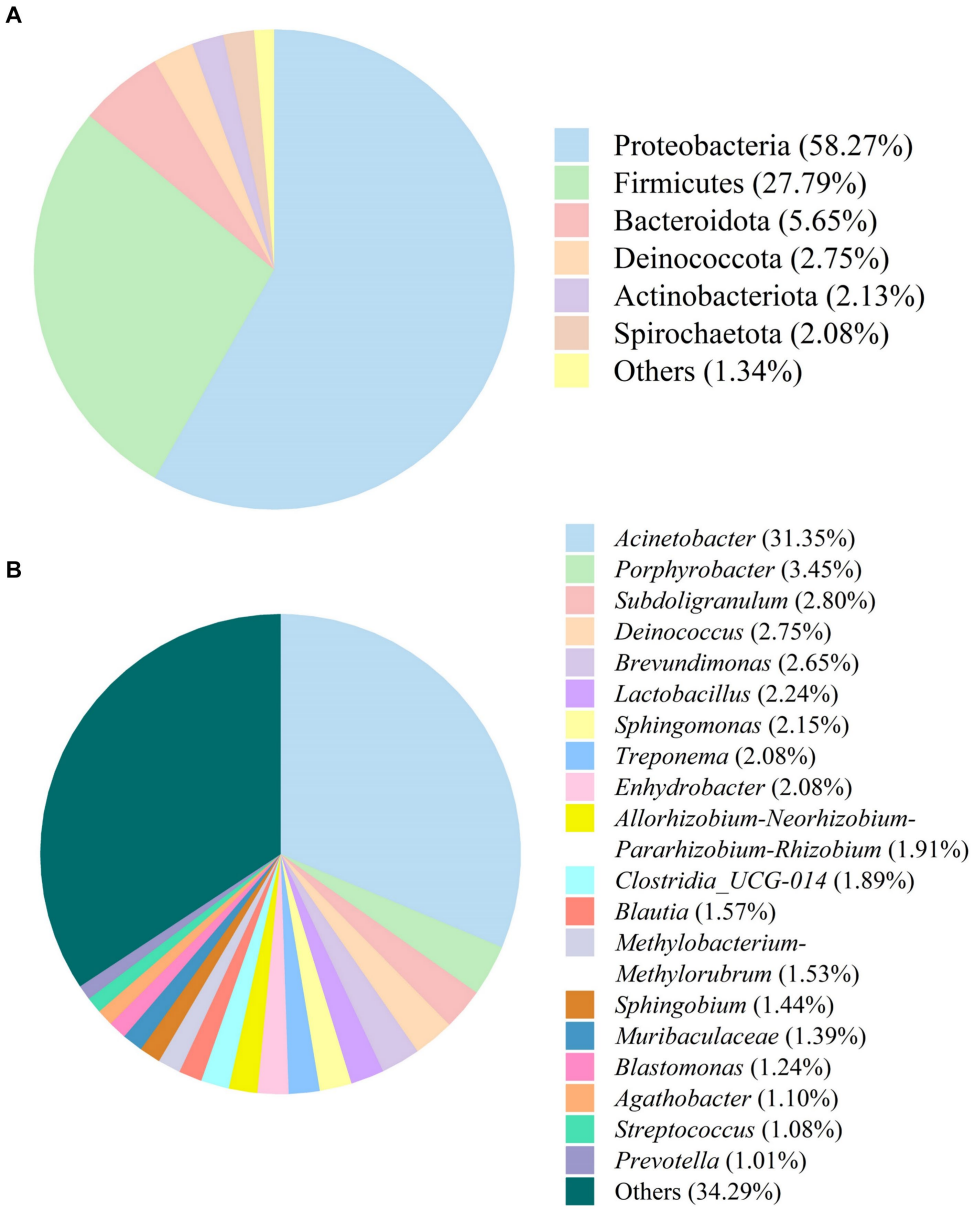
The bacteria composition of Shuanghuanglian fermentation liquor at the phylum **(A)** and genus **(B)** levels.

The bacterial composition in SFL is shown in Figure 6. Phyla with relative abundances ≤1.0% were not included in further analyses. Proteobacteria (58.27%) and Firmicutes (27.79%) were the most dominant phyla in SFL, followed by Bacteroidota (5.65%), Deinococcota (2.75%), Actinobacteria (2.13%), and Spirochaetota (2.08%) (Figure 6A). Genera with relative abundances ≤1.0% were excluded from further analysis. *Acinetobacter* (31.35%) was the most dominant genus in SFL, followed by *Porphyrobacter* (3.45%), *Subdoligranulum* (2.80%), *Deinococcus* (2.75%), *Brevundimonas* (2.65%), *Lactobacillus* (2.24%), *Sphingomonas* (2.15%), *Treponema* (2.08%), *Enhydrobacter* (2.08%), *Allorhizobium-Neorhizobium-Pararhizobium-Rhizobium* (1.91%), *Clostridia_UCG-014* (1.89%), *Blautia* (1.57%), *Methylobacterium-Methylorubrum* (1.53%), *Sphingobium* (1.44%), *Muribaculaceae* (1.39%), *Blastomonas* (1.24%), *Agathobacter* (1.10%), *Streptococcus* (1.08%), and *Prevotella* (1.01%) (Figure 6B).

**Figure 7:**
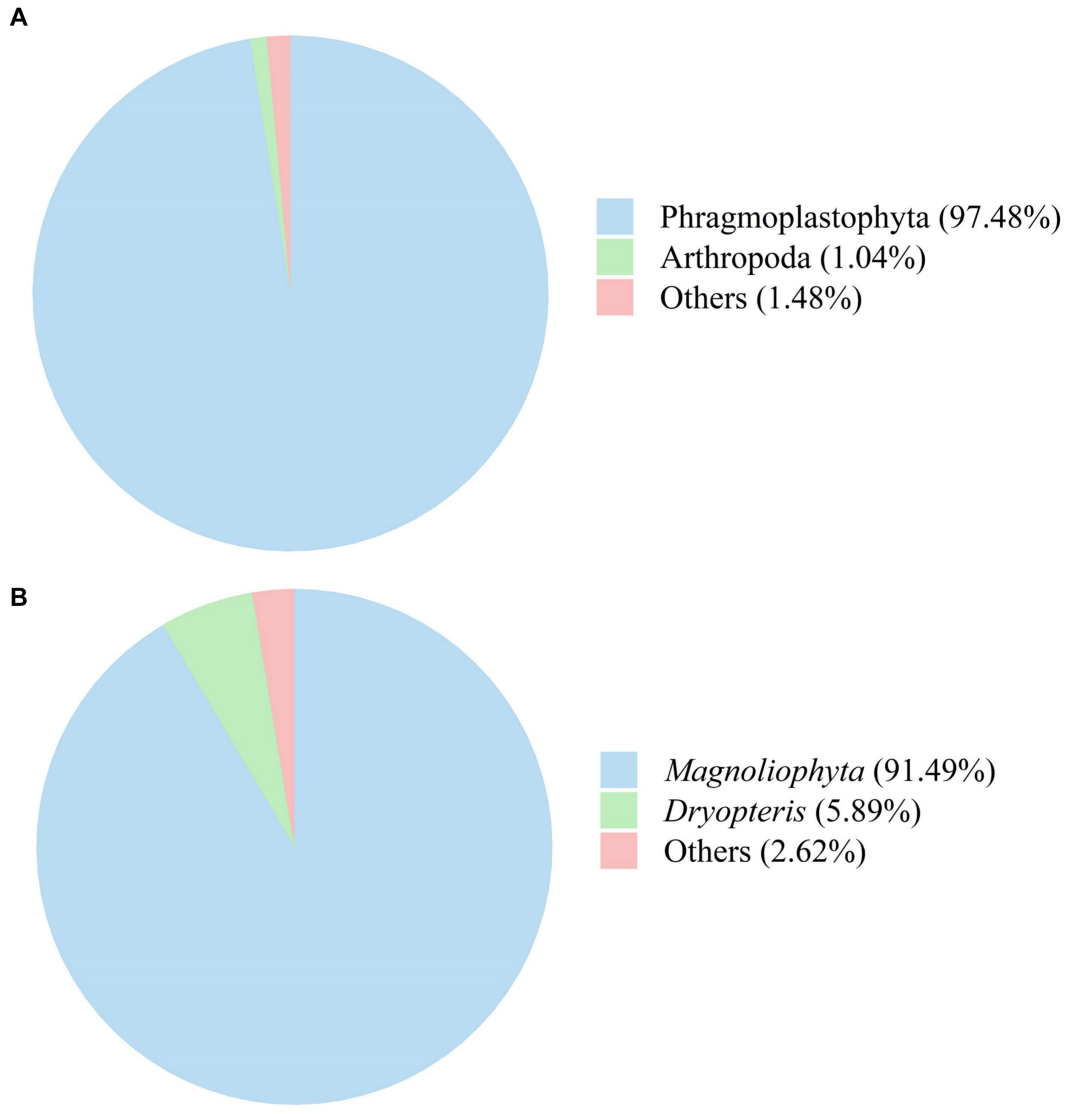
The fungi composition of Shuanghuanglian fermentation liquor at the phylum **(A)** and genus **(B)** levels.

The fungal composition of SFL is shown in Figure 7. Phyla with relative abundances ≤1.0% were not considered for further analyses. The relative abundances of Phragmoplastophyta and Arthropoda in SFL were 97.48 and 1.04%, respectively (Figure 7A). Genera with relative abundances ≤1.0% were not included in further analyses. The relative abundances of *Magnoliophyta* and *Dryopteris* in SFL were 91.49 and 5.89%, respectively (Figure 7B).

### 3.7 Laying performance and egg quality

The effects of SFL on laying performance and egg quality are presented in Table 8. Supplementation of 0.5 and 0.7% SFL in drinking water increased (*p* < 0.05) egg weight and laying rate and decreased (*p* < 0.05) feed-to-egg ratio compared with the control group. However, there was no significant difference (*p* > 0.05) in the ADFI among different groups.

**Table 8:** Effects of Shuanghuanglian fermentation liquor (SFL) on laying performance and egg quality of laying hens.

Item	SFL levels in water				SEM	*p*-values
	0%	0.3%	0.5%	0.7%		
Laying performance						
ADFI (g/d)	105.60	104.88	109.19	108.87	1.471	0.684
Egg weight (g)	51.26^c^	54.35^bc^	59.99^a^	57.90^ab^	1.095	0.007
Feed-to-egg ratio	2.07^a^	1.93^ab^	1.82^b^	1.88^b^	0.031	0.014
Laying rate (%)	70.85^b^	75.86^ab^	79.14^a^	78.26^a^	1.132	0.022
Egg quality						
Albumen height (mm)	4.50	4.80	5.00	4.83	0.101	0.404
Egg shape index	1.28	1.30	1.30	1.30	0.009	0.832
Eggshell thickness (mm)	0.36	0.36	0.37	0.37	0.004	0.797
Haugh unit	68.53^b^	72.17^a^	73.30^a^	72.20^a^	0.620	0.007
Yolk color	4.33	4.70	5.30	5.57	0.247	0.300

ADFI, average daily feed intake. Different letters indicate significant differences (*p* < 0.05).

Regarding egg quality traits, hens supplemented with 0.3, 0.5, and 0.7% SFL in drinking water had a higher (*p* < 0.05) Haugh unit than the control group, whereas there were no significant differences (*p* > 0.05) in albumen height, egg shape index, eggshell thickness, and yolk color among all groups.

## 4 Discussion

Shuanghuanglian has found to have anti-inflammatory, antibacterial, antiviral, and immunomodulatory effects (1–3). However, there is still a lack of research on the use of Shuanghuanglian in animal husbandry, particularly in the layer industry. In this study, fermentation was conducted with lignin-degrading bacteria (*Bacillus amyloliquefaciens-c4*) to enhance the bioactive components in Shuanghuanglian. This study optimized the fermentation conditions for Shuanghuanglian, investigated the compounds and microbial composition of SFL, and evaluated its effects on the production performance of laying hens. The results showed that supplementing SFL had positive effects on the production performance of laying hens. The bioactive components present in SFL, such as chlorogenic acid, baicalin, forsythin, myrtenol, 2-hexyn-1-ol, arsenous acid tris(trimethylsilyl) ester, 3(10)-caren-4-ol, and oxime-, methoxy-phenyl, may play a positive role in improving the production performance of laying. These components can also improve the immunity of laying hens due to their antibacterial, antiviral, and anti-inflammatory effects.

Chlorogenic acid, baicalin, and phillyrin are the main bioactive components of *Honeysuckle Flower*, *Baical Skullcap Root*, and *Fructus Forsythiae* (1), and have various biological effects such as antibacterial, antiviral, anti-inflammatory, anti-oxidative, and intestinal health-improving effects in laying hens (14–16). Thus, chlorogenic acid, baicalin, and phillyrin concentrations in SFL were assessed as representative indices to determine the optimal conditions for Shuanghuanglian. Moisture content, inoculation quantity, initial pH, particle size, rotational speed, fermentation time, and fermentation temperature are the main factors that could affect the concentrations of bioactive components present in Chinese medicine after fermentation (11, 17). Thus, in this study, we optimized these fermentation conditions. The concentrations of chlorogenic acid, baicalin, and phillyrin reached the highest values when the following parameters, including solid–liquid ratio, fermentation time, and particle size, were 1:7, 5 days, and 60 mesh. Therefore, the optimized fermentation conditions of solid–liquid ratio, fermentation time, and particle size for Shuanghuanglian were selected as a 1:7 SLR, 5 days, and 60 mesh through single factor experiments. However, Shuanghuanglian fermented with 3% inoculation had higher chlorogenic acid and baicalin concentrations, whereas fermented with 5% inoculation had higher baicalin and phillyrin concentrations in SFL. Shuanghuanglian fermented with 3% inoculation is more cost-effective than the 5% inoculation. Thus, this study used 3% inoculation for Shuanghuanglian fermentation. The optimized fermentation conditions of initial pH, temperature, and rotational speed were 6.99, 27.89°C, and 149.99 r/min, respectively, determined with response surface experiment. Considering the economical production, the optimal fermentation conditions of initial pH value, temperature, and rotational speed were set at 7, 28°C, and 150 r/min, respectively. Finally, the optimized fermentation conditions for Shuanghuanglian were selected as a 1:7 SLR, inoculation quantity 3%, initial pH 7, 60 mesh particle size, rotational speed 150 r/min, and 28°C fermentation for 5 days through single factor and response surface experiments.

We further determined the composition of SFL and identified various compounds with important biological efficacy. Among these compounds, myrtenol has been reported to have antibacterial, antibiofilm, anti-inflammatory, and antinociceptive effects (18, 19). In the present study, SFL contained higher myrtenol content, indicating that SFL may reduce disease incidence and damage to poultry. In addition, 2-hexyn-1-ol contributes to the antibacterial activity (20), and arsenous acid tris(trimethylsilyl) ester is a flavor-related compound (21, 22). Similar to the present study, arsenous acid tris(trimethylsilyl) ester and 3(10)-caren-4-ol have been detected in various Chinese herbal medicines in previous studies and exhibited antibacterial effects (23–26). Moreover, oxime-, methoxy-phenyl is also a flavor-related compound (27) and has been found to have antitumor and anticancer effects (28). Therefore, supplementing SFL to laying hens may increase disease resistance and feed intake, thereby enhancing production performance.

Proteobacteria and Firmicutes were the most abundant phyla in SFL, which is consistent with the bacterial phyla composition of Chinese herbal medicines fermented by *Bacillus amyloliquefaciens-c4* (11). *Acinetobacter* was the most abundant genus detected in SFL and can produce various umami peptides (29). Thus, a higher relative abundance of *Acinetobacter* in SFL may increase the feed intake in poultry. However, there was no significant change in the abundance of *Bacillus* in the present study despite inoculation with 3% *Bacillus amyloliquefaciens-c4*, which is inconsistent with a previous study (11). This inconsistency may be due to *Bacillus amyloliquefaciens-c4*, being a lignin-degrading bacterium, attaching primarily to the cell wall of Shuanghuanglian rather than releasing it to SFL. This statement is also supported by the more disrupted surface morphology of the *Honeysuckle Flower*, *Baical Skullcap Root*, and *Fructus Forsythiae* after fermentation in the present study. In addition, Phragmoplastophyta was the main fungal phylum, and *Magnoliophyta* was the main fungal genus in SFL. The findings are consistent with the findings of Yi et al. (11), who reported that Chinese herbal medicines fermented with *Bacillus amyloliquefaciens-c4* had higher abundances of Phragmoplastophyta and *Magnoliophyta*. The genera *Magnoliophyta* and *Dryopteris* belong to the phylum Phragmoplastophyta and exist widely in the air and soil environments (30), which may explain their abundance in SFL.

To evaluate the effects of SFL, different levels of SFL were supplemented with drinking water in laying hens. The results showed that 0.5 and 0.7% SFL supplementation increased egg weight and laying rate while decreasing the feed-to-egg ratio of laying hens. The increased production performance of laying hens is possibly due to the antibacterial and anti-inflammatory effects of SFL. In addition, supplementation of SFL increased the Haugh units of eggs, indicating that the bioactive components present in SFL improved the albumen and storage quality of eggs (31, 32). Wang et al. (14) also found that supplementation of *Honeysuckle Flower* and *Baical Skullcap Root* extracts in laying hens’ diet alleviated intestinal disorders and performance impairment caused by *Salmonella pullorum* infection. Interestingly, SFL supplementation had no effects on the ADFI of laying hens, although it included various flavor-related compounds, which may have been caused by the addition of SFL in drinking water rather than in the diet. Furthermore, supplementation of 0.3% SFL had no effects on the laying performance of hens, indicating that SFL supplementation in drinking water should be at least 0.5%. Overall, the findings indicate that SFL supplementation had beneficial effects on the production performance of laying hens, especially when supplementation reached 0.5%.

## 5 Conclusion

The optimal fermentation conditions for Shuanghuanlian were determined as follows: a SLR ratio of 1:7, 3% inoculation quantity, fermentation at 28°C for 5 days, initial pH of 7, a 60 mesh particle size, and a rotational speed of 150 r/min. These conditions are crucial in ensuring consistent product quality and composition for future testing and application. Supplementing at least 0.5% SFL in drinking water had positive effects on the production performance of laying hens, including increased egg weight, laying rate, and Haugh unit and decreased feed-to-egg ratio, which indicate that SFL based on the optimized fermentation conditions could positively influence the production performance of laying hens. Further in-depth studies are necessary to explore the long-term effects of SFL, the mechanisms of bioactive components in SFL, and the combination effects with other feed additives or drugs on the production performance of laying hens. Overall, these findings provide a solid foudation for further investigation into the use of Shuanghuanglian as a natural feed addition to improve the productivity and sustainability of egg production.

## Data Availability

The data presented in the study are deposited in the Figshare repository, accession number is doi: 10.6084/m9.figshare.27050017.v1.
